# Determining amplitude and tilt of a lateral force microscopy sensor

**DOI:** 10.3762/bjnano.12.42

**Published:** 2021-06-01

**Authors:** Oliver Gretz, Alfred J Weymouth, Thomas Holzmann, Korbinian Pürckhauer, Franz J Giessibl

**Affiliations:** 1 Institute of Experimental and Applied Physics, Department of Physics, University of Regensburg, 93053 Regensburg, Germany

**Keywords:** frequency-modulation atomic force microscopy, lateral force microscopy, amplitude calibration, tilt estimation

## Abstract

In lateral force microscopy (LFM), implemented as frequency-modulation atomic force microscopy, the tip oscillates parallel to the surface. Existing amplitude calibration methods are not applicable for mechanically excited LFM sensors at low temperature. Moreover, a slight angular offset of the oscillation direction (tilt) has a significant influence on the acquired data. To determine the amplitude and tilt we make use of the scanning tunneling microscopy (STM) channel and acquire data without and with oscillation of the tip above a local surface feature. We use a full two-dimensional current map of the STM data without oscillation to simulate data for a given amplitude and tilt. Finally, the amplitude and tilt are determined by fitting the simulation output to the data with oscillation.

## Introduction

**Figure 1:**
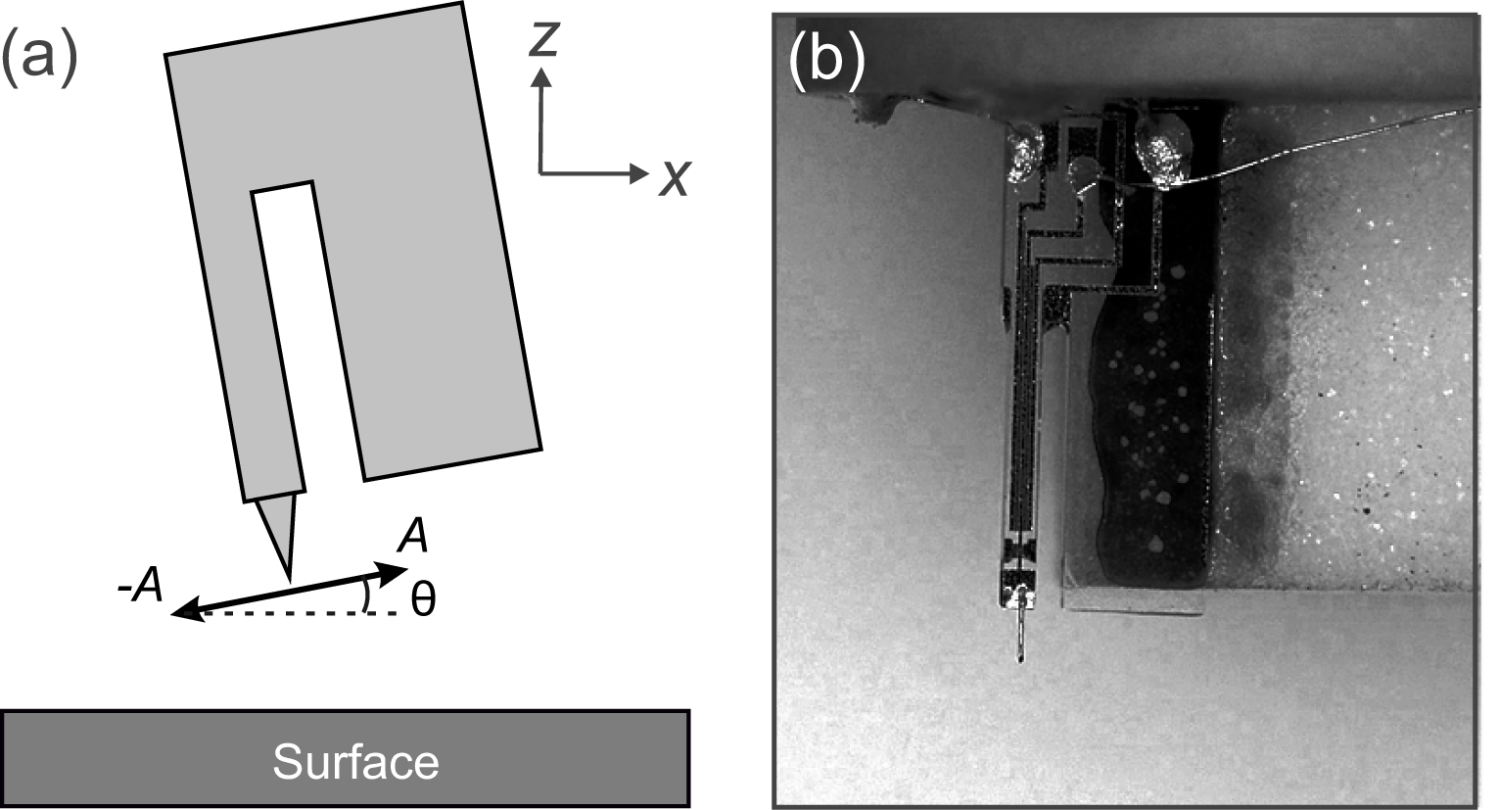
(a) Sketch of the qPlus sensor in LFM orientation with amplitude *A* and the sensor tilt θ. (b) Photograph of a qPlus sensor glued perpendicularly on a sensor holder shown by the white substrate.

Frequency-modulation atomic force microscopy (AFM) is a non-contact atomic force microscopy technique where the frequency shift (Δ*f*) of an oscillating tip is detected [1]. The frequency shift is a measure of the total force gradient acting on the tip, which includes both long-range and short-range contributions. A typical experimental setup is to study an isolated surface feature, for instance, a defect or an adsorbate, on a flat terrace. In case of “normal” AFM, where the tip oscillates perpendicular to the surface, long-range forces including electrostatic and van der Waals forces contribute to the measured Δ*f* signal, which have to be subtracted in order to isolate the short-range contributions from the surface feature [2]. If the cantilever is rotated by 90° so that the tip oscillates lateral to the surface, long-range forces with large vertical components do not contribute to the Δ*f* signal [3]. This microscopy technique is called lateral force microscopy (LFM) (Figure 1). One advantage to LFM is that it is highly sensitive to short-range interactions. A drawback is that it is not a suitable technique for approaching a sample or determining the sample tilt. Here a complementary technique such as STM (used in our setup) or biaxial AFM with normal force detection is required.

Experimentally, there are several methods for performing frequency-modulation lateral force microscopy, what we refer to as LFM in this manuscript. In 2002, Pfeiffer and co-workers excited a silicon cantilever in the first torsional mode [4]. This has been used to achieve atomic resolution of a sample that is laterally stiff and vertically soft [5]. It has also been used under ultrahigh-vacuum conditions [6] as well as in liquid to yield atomic resolution [7]. Also in 2002, Giessibl and co-workers performed LFM using a qPlus sensor as shown in Figure 1b [8]. In our group, we have used this method to quantify molecular stiffness at low temperature [9] and to evaluate the potential energy landscape above a molecule at room temperature [10]. More recently, we used LFM with a CO-terminated tip to investigate the internal structure of a molecular adsorbate [11–12]. Moreover, other methods, including the use of a long tip on a qPlus sensor that oscillates laterally at a higher flexural mode are also possible [13].

In LFM or normal AFM, the recorded frequency shift Δ*f* is related to the force gradient *k*_ts_ in the direction of the tip oscillation. For a sensor oscillating in the *x*-direction,


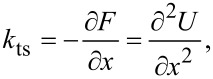


where *F* is the component of force in the *x*-direction and *U* is the potential energy. In general, the relevant force gradient at a spatial coordinate (*x*, *z*) for a tip oscillating at an angle θ with respect to the *x*-direction is:

[1] $$k_{\text{ts}}\left(x,z\right)=\frac{\partial^{2}U\left(x,z\right)}{\partial x^{2}}\cos^{2}\theta +\frac{\partial^{2}U\left(x,z\right)}{\partial z^{2}}\sin^{2}\theta .$$

The frequency shift is related to the sensor parameters and the weighted average of the force gradient over the tip oscillation, ⟨*k*_ts_⟩(*x*_0_, *z*_0_), where *x*_0_ and *z*_0_ define the average tip position over one oscillation cycle [14]:

[2] $$\Delta f\left(x_{0},z_{0}\right)=\frac{f_{0}}{2k}\langle k_{\text{ts}}\rangle \left(x_{0},z_{0}\right).$$

Here, *f*_0_ is the resonance frequency of the sensor away from the surface and *k* is the stiffness of the sensor. The weighted average must also take into account the direction of the tip oscillation:

[3] $$\langle k_{\text{ts}}\rangle \left(x_{0},z_{0}\right)=\frac{2}{\pi A^{2}}\int_{-A}^{A}k_{\text{ts}}\left(x_{0}-q\cos\theta ,z_{0}-q\sin\theta \right)\sqrt{A^{2}-q^{2}}\text{d}q,$$

where *A* is the oscillation amplitude. Extracting force and potential energy from the measured Δ*f* is a complex inversion problem requiring deconvolution. Several deconvolution methods include a matrix inversion method developed by Giessibl [15], a Laplace transform method developed by Sader and Jarvis [16], and a Fourier method developed by Seeholzer and co-workers [17]. All of these methods require the knowledge of the oscillation amplitude *A* of the cantilever.

Amplitude determination means determining a calibration factor that relates the recorded amplitude signal of the oscillation in volts to the real oscillation amplitude in meters. At room temperature the thermal excitation of the sensor can be used to calibrate the amplitude [18]. For low temperatures, another method has to be used since the thermal energy to excite the sensor is very small and mechanical vibrations can dominate the excitation [19]. For low-temperature LFM, the lattice of the substrate can be used to calibrate the amplitude if the periodicity of the lateral features is known [20]. For electrically excited piezoelectric-based sensors, the energy input required to maintain the oscillation amplitude constant can be measured to calculate the calibration factor [21]. Besides these experimental methods, the amplitude can be also calibrated by calculating the electro-mechanical properties of the cantilever [22]. This theoretical method, however, does not take the real geometry of the sensor and electrodes into account. At low temperatures, the most common method is to use STM to calibrate the amplitude, provided that STM is available and that the sample is conducting. This method is often used for normal AFM, where the cantilever oscillates vertically to the surface, and the current is related to the vertical position *z* of the tip above the sample, via *I* = *I*_0_exp(-2κ*z*), where *I*_0_ is the current at *z* = 0 m and κ is the decay constant [23]. For non-conducting surfaces Δ*f* spectra with different oscillation amplitudes can be used [23].

### Comparing the effect of tilt on normal versus lateral force microscopy

In addition to the amplitude, the tilt θ of a LFM sensor is of great importance. Usually, θ is ignored in normal AFM experiments because it has a smaller effect on the observed values of Δ*f*. This can be seen by modelling ⟨*k*_ts_⟩ of a normal AFM sensor and comparing it to the signal of a LFM sensor. The position of the tip at time *t*, as it oscillates around a point *x*_0_, *z*_0_, is given by

[4] $$x\left(t,x_{0}\right)=A\cos\left(2\pi ft\right)\cos\theta +x_{0},$$

[5] $$z\left(t,z_{0}\right)=A\cos\left(2\pi f\right)\sin\theta +z_{0},$$

where *t* is the time, θ is the tilt of the sensor as defined above and *f* = *f*_0_ + Δ*f*. We model the interaction between the tip and a surface feature as a Morse potential:

[6] $$U\left(r\right)=E_{\text{B}}\left[\exp\left(-2\frac{r-\sigma }{\lambda }\right)-2\exp\left(-\frac{r-\sigma }{\lambda }\right)\right],$$

where *E*_B_ is the binding energy, σ is the equilibrium distance, and λ is the decay length. The position of the tip, *x* and *z*, yield the distance to the surface feature *r* = (*x*^2^ + *z*^2^)*^1/2^* (i.e., the feature is located at (0, 0)).

**Figure 2:**
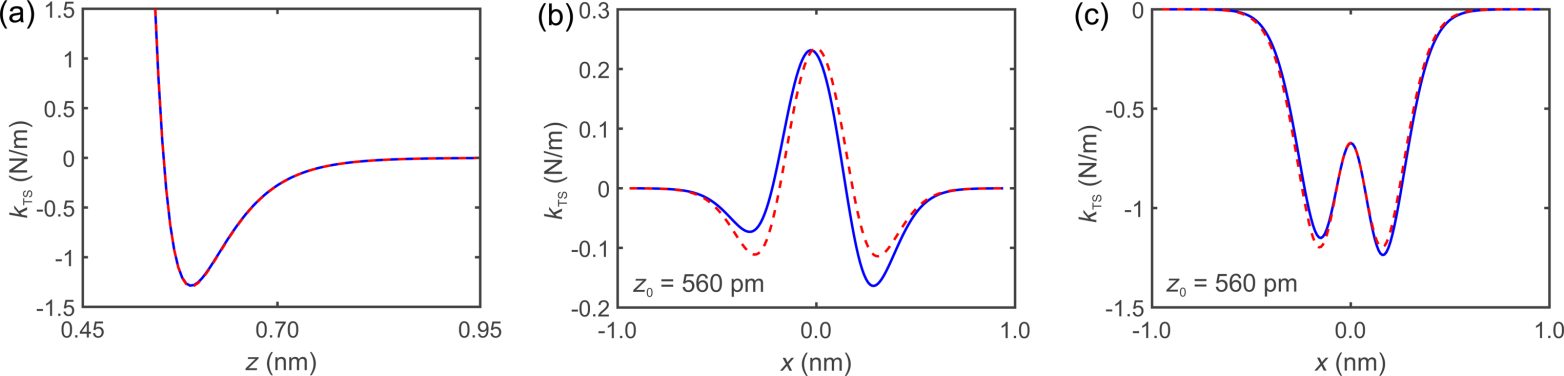
Simulated Morse potential interaction for a LFM setup and comparison to normal AFM. θ is defined by Equation 1. (a) Simulated force gradient ⟨*k*_ts_⟩ of a vertically oscillating tip above an atom with θ = 86° shown by the blue solid curve and without tilt (θ = 90°) shown by the red dashed curve. The atom was simulated by a Morse potential. (b) ⟨*k*_ts_⟩ of a laterally oscillating tip above an atom with sensor tilt θ = 4° shown by the blue solid curve and without (θ = 0°) shown by the red dashed curve at height *z*_0_ = 560 pm. The height *z*_0_ is defined according to Equation 5. The ratio of the difference between the minima of the curve with tilt and without tilt to the overall curve without tilt is 14.2%. (c) ⟨*k*_ts_⟩ of a vertically oscillating tip following the path of the laterally oscillating tip at the same height with a tilt (θ = 86°, blue solid curve) and without a tilt (θ = 90°, red dashed curve). The ratio of the difference between the minima of the curve with tilt and without tilt to the overall curve without tilt is 3.3%.

We used the following parameters for the Morse potential: *E*_B_ = 100 meV, σ = 500 pm and λ = 50 pm. We first calculated the *z*-dependence of ⟨*k*_ts_⟩ for a tip with no tilt oscillating vertically above the center of the adsorbate. For θ, as defined in Figure 1a, being 90°, the calculated values of ⟨*k*_ts_⟩ are shown in Figure 2a (red dashed curve).

We also calculated ⟨*k*_ts_⟩ with a small tilt from the vertical so that θ = 86°, shown in Figure 2a by the blue solid curve. The similarity of the two curves shows that the tilt has little influence on normal AFM data.

We then calculated the *x*-dependence of ⟨*k*_ts_⟩ for a LFM tip with no tilt, where θ = 0°, shown in Figure 2b by the red dashed curve at *z*_0_ = 560 pm, and contrasted it to the LFM signal with a tilt of θ = 4°, shown in Figure 2b by the blue solid curve. The tilted LFM signal is strongly asymmetric with a lower local minimum and a higher one. Also, the peak at *x* = 0 pm is slightly shifted.

Figure 2c shows a vertically oscillating tip following the tip path of the laterally oscillating tip from Figure 2b at the same height with θ = 90° displayed by the red dashed curve and with θ = 86° displayed by the blue solid curve. The ratio of the difference between the minima of the curve with tilt and the curve without tilt to the overall curve without tilt is 3.3%, in contrast to the ratio of the LFM curves in Figure 2b, which is 14.2%. This larger difference between the LFM curves shows that sensor tilt is more visible in LFM data.

### Effect of amplitude and tilt on the STM signal in LFM

**Figure 3:**
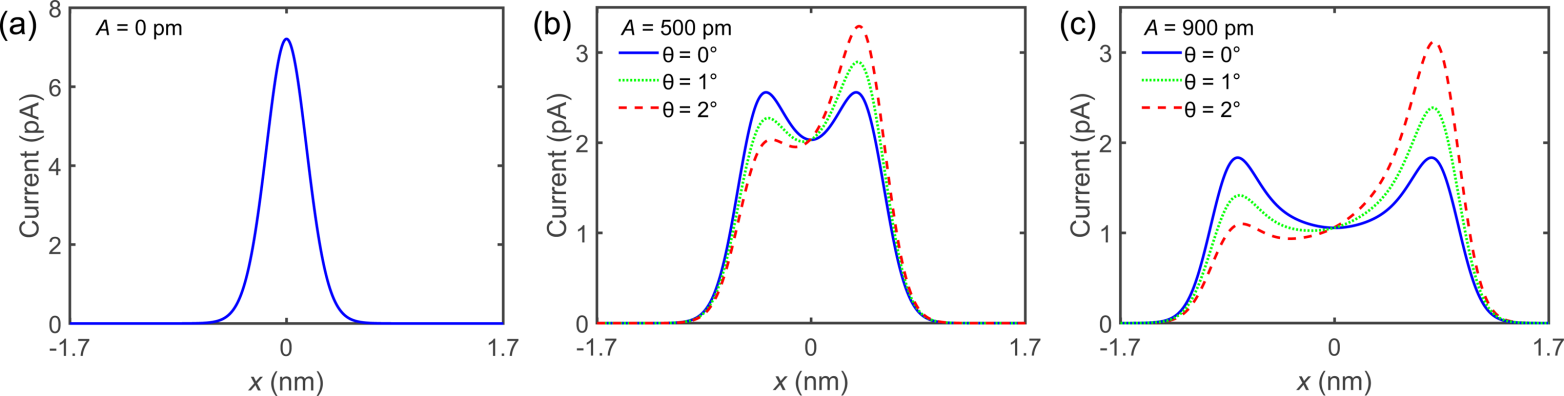
Simulating the effect of a laterally oscillating tip with different amplitudes and tilt angles on the STM signal. (a) With no oscillation. (b) With an amplitude of 500 pm and θ = 0°, shown by the blue solid curve, θ = 1°, shown by the green dotted curve, and θ = 2°, shown by the dashed red curve. With increasing oscillation amplitude, two peaks evolve, which differ in height in dependence on the tilt angles. (c) With an amplitude of 900 pm and θ = 0°, displayed by the blue solid curve, θ = 1°, displayed by the green dotted curve, and θ = 2°, displayed by the red dashed curve. With a higher oscillation amplitude the difference between the two peaks increases.

In the following the influence of tip oscillation and tip tilt on the current signal is demonstrated for LFM. Due to the bandwidth of the STM channel, the recorded signal ⟨*I*⟩ is the average of the current over the motion of the tip [24]:

[7] $$\langle I\rangle \left(x_{0},z_{0}\right)=\frac{1}{T}\int_{0}^{T}I\left(x(\tau ,x_{0}),z(\tau ,z_{0})\right)\text{d}\tau ,$$

where *T* = 1/*f* is the period. *I*(*x*, *z*) is the tunneling current at time τ at the coordinates *x* and *z* of the tip described by Equation 4 and Equation 5. Consider a surface feature that appears with no oscillation as a Gaussian curve as shown in Figure 3a. With large oscillation amplitudes (*A >* σ) the current curve becomes wider with two maxima, as shown by the blue solid curve in Figure 3b for an oscillation amplitude of *A* = 500 pm. At an amplitude of 900 pm the distance between the maxima increases as shown by the blue solid curve in Figure 3c. When we vary θ and set it to, for example, θ = 1°, the two local maxima become vertically shifted, one higher and the other one lower as depicted by the green dotted curve in Figure 3b and in Figure 3c. With a tilt of θ = 2°, the two local maxima become even more separated as illustrated by the red dashed curve in Figure 3b,c. The differences in current of the local maxima are related to the tilt of the sensor and the horizontal distance depends on the oscillation amplitude.

In this paper we present a method to calibrate the amplitude and determine the tilt of the LFM sensor. The method is based on collecting STM data of a surface feature both without and with tip oscillation, as was proposed in [20]. The data without oscillation is used as input to a simulation that calculates expected STM data with oscillation as a function of *A* and θ. *A* and θ are then determined by fitting the calculated data to the experimental data. A two-dimensional current map is used.

## Experimental

Measurements were performed with a low-temperature STM/AFM system (CreaTec Fischer GmbH, Berlin, Germany) operating in ultra-high vacuum at 5.6 K equipped with a qPlus sensor [25]. The sensor was equipped with an etched tungsten tip, which was repeatedly poked into a Cu(111) surface to generate well-defined tip apex configurations. Cu(111) was cleaned by standard sputtering and annealing cycles. Single iron adatoms were evaporated with a custom-built evaporator onto the cold sample. Carbon monoxide (CO) was leaked in at a partial pressure of 5 × 10^−8^ mbar for 5 min.

## Results and Discussion

### Determining *A* and θ with a 2D current map

**Figure 4:**
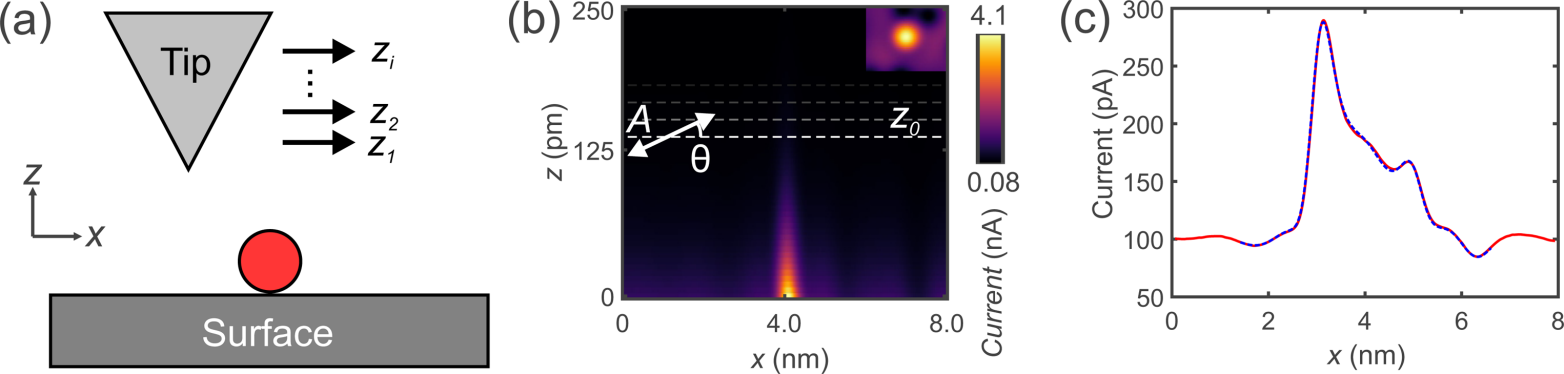
Two-dimensional (2D) current map without oscillation and calculated and recorded curve with oscillation. (a) Sketch of the tip taking a 2D dataset of a surface feature (red circle) at different heights *z_i_*. (b) 2D current map of an iron adatom on Cu(111) taken with a metal tip. The dashed white line indicates the current line at *z*_0_ at which the 2D current map is used to calculate the curve with oscillation. The inset shows a constant current STM image of an iron adatom on Cu(111), which is used for the 2D current map. (c) Line profile of a constant-height scan of an iron adatom on Cu(111) with oscillation shown by the red solid curve. The fit uses the 2D current map yielding an amplitude *A* = 1050 pm ± 2% and a sensor tilt of θ = 1.59° ± 2% shown by the blue dashed curve.

In the following, a method to determine *A* and θ is presented. As shown before, *A* and θ influence the shape of the average current signal, ⟨*I*⟩. Initially, the current *I* above a surface feature is recorded without oscillation, as sketched in Figure 4a. In the next step, current data acquired with oscillation can be simulated at a given height *z*_0_, 
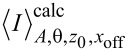
 using Equation 7, with *A*, θ, and *x*_offset_ as parameters. By varying *z*_0_, *A*, θ, and *x*_offset_, we fit 
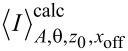
 to the measured ⟨*I*⟩ to determine the amplitude and tilt.

To do this we first acquired a full two-dimensional current map without oscillation, as sketched in Figure 4a, to measure *I*(*x*, *z*). Figure 4b shows *I*(*x*, *z*) above a single iron adatom on Cu(111) taken with a metal tip. The similarity with the simulated curves shown in Figure 3 can be seen. However, small discrepancies are visible. The reason for the discrepancies are Cu(111) surface states and nearby CO molecules, which were captured in the data. The inset in Figure 4b shows an STM image of a single iron adatom [26]. The red solid curve in Figure 4c shows the current profile ⟨*I*⟩ along a line in the *x*-direction over a single iron adatom on Cu(111) with tip oscillation. The blue dashed curve is the fitted 
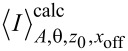
, yielding *A* = 1050 pm ± 2% and θ = 1.59° ± 2%. The difference between ⟨*I*⟩ and 
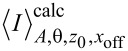
 was 0.48%.

The tip tilt angle is defined by the tilt relative to the flat sample, as shown in Figure 1a. Different regions on the sample can have different sample tilts, which would change the relative tip tilt. To account for this, the sample tilt must be accounted for before measuring.

To efficiently determine the best-fit parameters, an automated fitting algorithm was programmed. This algorithm minimizes the least squares error between the calculated curve and the recorded curve with oscillation. A common problem when determining the least squares error is to find the global minimum (best fit). Tests with different starting values revealed that the error landscape has many local minima. These local minima have higher least squares errors than the global minimum, which can be fitted to a much lower least squares error. By defining a sufficiently low error limit *e*_LIM_, many of the local minima with higher least squares error can be ruled out. A good start is a value of *e*_LIM_ = 0.5% to find parameters that can be used as starting parameters for further runs of the software. For the consecutive runs, *e*_LIM_ can be reduced by 0.1% for each run until an acceptable fit is found. Note that the program will not converge to a solution if *e*_LIM_ is too small because of noise and factors such as drift.

Another problem is the choice of starting values. To try different combinations with equal probability, random starting values within a definable interval were generated. Care has to be taken at the definition of the intervals. Our tests showed that these intervals have to cover the final fitted values for *A*, θ, and *z*_0_ to find the best fit. Therefore, reasonably chosen starting values are necessary. An estimate of the amplitude can be made by considering data with oscillation of a single feature, as sketched in Figure 3. The data will show the feature spread by a lateral extent of approximately 2*A*. The relative heights of the feature are indicative of the tilt, which can be estimated by comparing to Figure 3b,c.

**Figure 5:**
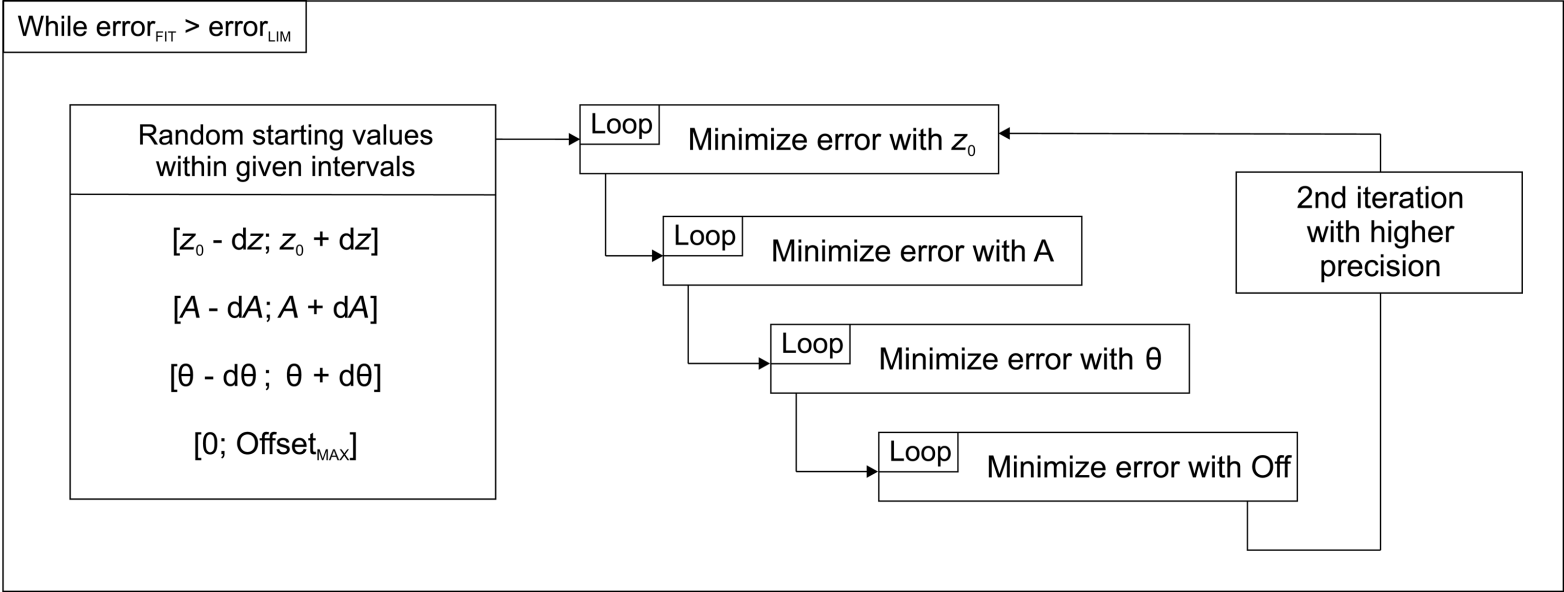
Structure of the fitting algorithm to avoid a local minimum. Random starting values within definable interval are used to start the first iteration to minimize the least squares error for *z*_0_, *A*, θ, and “offset” until the total error *e*_FIT_ *< e*_LIM_.

Figure 5 shows the structure of the fitting algorithm. The outer “while” loop starts the fitting with the random starting parameters as long as *e*_FIT_ *> e*_LIM_.

The parameters are changed in the direction in which the least squares error gets smaller. The order in which the parameters are changed has significant impact on the convergence of the error. The *x*_offset_ parameter is the lateral offset of the calculated curve to the data taken with oscillation. Since a variation of *z*_0_ leads to a higher variation of the least squares error than a variation of *A*, θ, or *x*_offset_, *z*_0_ is determined first, followed by *A*, θ, and then *x*_offset_, as it is shown in Figure 5. After this iteration, the whole loop is started over again with a higher precision and *e*_FIT_ is calculated.

The numeric implementation requires a discretization of the oscillation cycle dividing the period *T* into a number of points. Lower errors can be obtained with a higher number of discretization points up to a certain limit. Our tests showed that a number of discretization points of 250 is a good compromise between fitting accuracy and computation time.

The fitting algorithm yields a very high repetitive accuracy if *e*_LIM_ is low enough. To test how robust our algorithm is, we performed five fits using starting values in a range of ±30% of the actual values. This led to almost equal values determined by the algorithm for *A* and θ, depending on the set error. In the case of our fits, we used *e*_LIM_ = 0.1%, which resulted in a spread of *A* of 0.2%.

**Figure 6:**
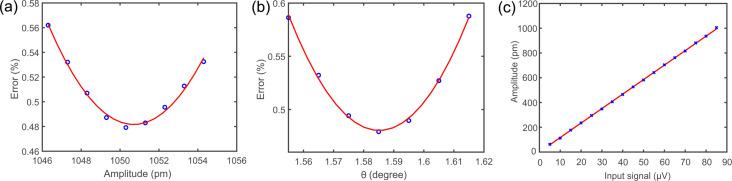
Error as a function of *A* and θ. (a) The blue circles show the calculated error for varying amplitudes. The other parameters θ, *z*_0_, and *x*_offset_ were kept constant. The red curve shows a parabolic fit through the points to estimate the uncertainty of the fitted *A* and yielded a value of 0.002 pm. (b) The calculated error for varying θ shown by the blue circles. The other parameters *A*, *z*_0_, and *x*_offset_ were kept constant. The red curve shows a parabolic fit through the points to estimate the uncertainty of the fitted θ and yielded a value of 0.001°. (c) Amplitude as a function of the drive signal.

To estimate the uncertainty of the fitted values of *A* and θ we calculated the error as a function of *A* and θ as shown by the blue circles in Figure 6a,b. In Figure 6a the error as a function of the amplitude is shown. The other parameters θ, *z*_0_, and *x*_offset_ were kept constant. The fitted amplitude value of 1050 pm is indeed a minimum, since the error around this value increases. To calculate the uncertainty we fitted a parabolic function according to *E*(*A*) = *a*(*A* − *b*)^2^ + *c* to the data points. *a*, *b*, and *c* are the fitting parameters of the parabola. *b* represents the location of the minimum. The Matlab function “fit” outputs the variation of the fitting parameters with confidence bounds of 95%. From this confidence bounds the uncertainty for *b* was calculated yielding a very small value of 0.002 pm for the amplitude.

The same procedure was applied to estimate the uncertainty for θ. The blue circles in Figure 6b show the calculated error as a function of θ. The other parameters *A*, *z*_0_, and *x*_offset_ were kept constant. The error increases around the fitted minimum of 1.59°. The red curve shows a parabolic fit according to the equation *E*(θ) = *g*(θ − *h*)^2^ + *i*. This yielded also a very small uncertainty for *h* of 0.001°.

The method inherently yields a very small uncertainty for both the amplitude and tilt. However, it relies upon the (*x*, *y*)-calibration of the microscope, which we assume to the be the largest source of uncertainty. The position calibration typically has a precision of the order of a few percent, which is why we propose an uncertainty of 2%. A similar argument for accuracy holds for the tilt estimate. It relies on the calibration of *x*, *y*, *z*, and sample tilt; again, we estimate an uncertainty of 2%.

Figure 6c shows the amplitude of the oscillation as a function of the drive signal. It is linear, meaning that the calibration amplitude we determine for large amplitudes of the order of 1 nm is also valid for amplitudes under 100 pm, where we typically acquire high-resolution data.

**Figure 7:**
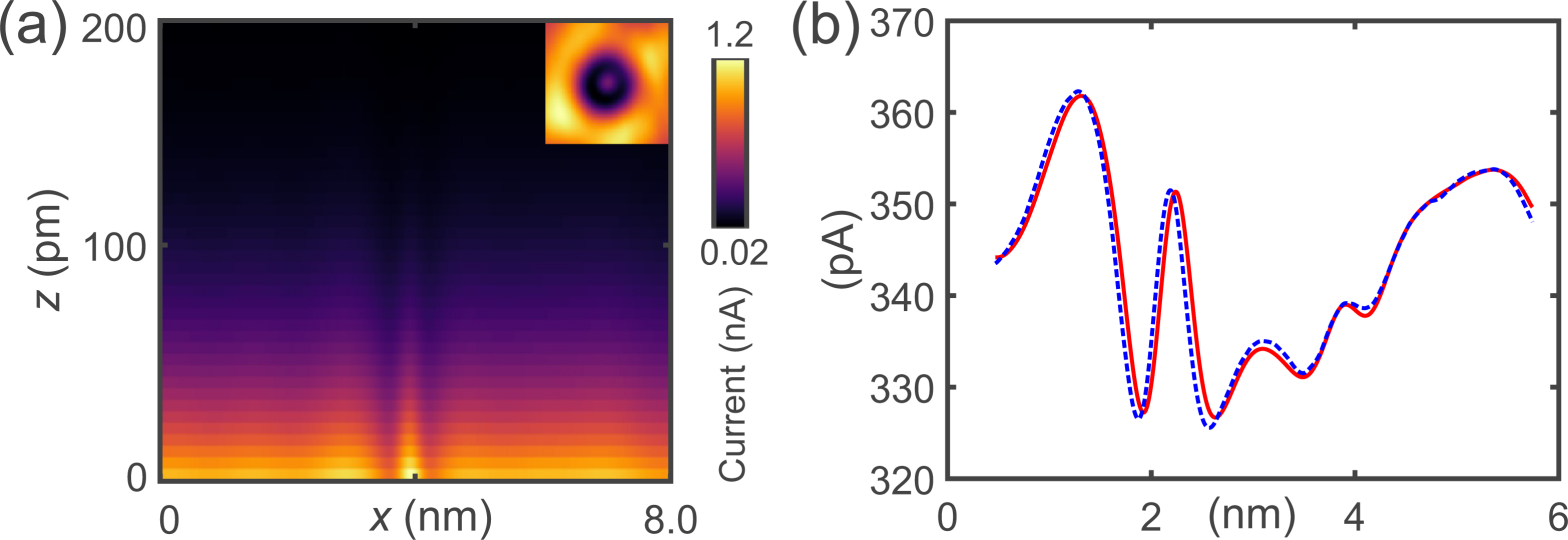
Two-dimensional (2D) current map without oscillation and calculated and recorded curve with oscillation. (a) 2D current map of a carbon monoxide molecule (CO) on Cu(111) taken with a CO molecule adsorbed on the tip (CO tip). The dashed white line indicates the current line at *z*_0_ at which the 2D current map is used to calculate the curve with oscillation. The inset shows a constant current STM image of a CO on Cu(111), which is used for the 2D current map. (b) Line profile of a constant-height scan of a CO molecule on Cu(111) with a CO tip with oscillation shown by the red solid curve. The fit uses the 2D current map yielding an amplitude *A* = 890 pm ± 2% and sensor tilt of θ = 2.00° ± 2% shown by the blue dashed curve.

To demonstrate that this method can be applied to more complex systems, calibration data was taken of a CO molecule on Cu(111) with a CO tip. When lateral forces act on the CO molecules on the tip and the surface, they tend to act as a torsional spring and bend [27–28]. While this makes the signal more complex than that over the Fe adatom (compare Figure 7b to Figure 4c), the CO bending does not affect the measurements. In general, if CO bending occurs, it is present in the data both with oscillation and without as the CO bends faster than the cantilever moves. At the heights where we performed the amplitude calibration, we did not observe a LFM signal, meaning that the lateral forces were insignificant. Also, the excitation frequency of the frustrated translational mode is in the terahertz range [29–30] and is not excited by the tip, which oscillates in the kilohertz range.

Figure 7a shows the *I*(*x*, *z*) current map without oscillation. In the inset, an STM image of a CO molecule with a CO tip is shown [31]. The red solid curve in Figure 7b shows the current profile ⟨*I*⟩ along a line in *x*-direction over a CO molecule on Cu(111) with a CO tip. The dashed blue curve in Figure 7b is 
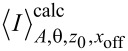
 and yielded *A* = 890 pm ± 2% and θ = 2.00° ± 2%.

## Conclusion

A method of determining the oscillation amplitude and tilt of a LFM sensor was presented by analyzing the tunneling current above a surface feature. The method requires a 2D current map without tip oscillation and an isolated line profile with oscillation. It fits a calculated averaged current curve that considers the tip oscillation to a constant height current curve with oscillation to determine *A* and θ. The method can be applied, in principle, to any surface feature such as, for example, a commonly used PTCDA molecule or a surface defect. The fitting of the parameters for the 2D current map method was done by a fitting algorithm written in MATLAB, and details of the algorithm were explained. A MATLAB file is included in Supporting Information File 1.

## Supporting Information

File 1 MATLAB file of the fitting algorithm.
